# Different methods for volatile sampling in mammals

**DOI:** 10.1371/journal.pone.0183440

**Published:** 2017-08-25

**Authors:** Marlen Kücklich, Manfred Möller, Andrea Marcillo, Almuth Einspanier, Brigitte M. Weiß, Claudia Birkemeyer, Anja Widdig

**Affiliations:** 1 Junior Research Group of Primate Kin Selection, Department of Primatology, Max-Planck-Institute for Evolutionary Anthropology, Leipzig, Germany; 2 Behavioural Ecology Research Group, Institute of Biology, University of Leipzig, Leipzig, Germany; 3 Institute and Out-patient Clinic of Occupational Medicine, RWTH Aachen University, Aachen, Germany; 4 Research Group of Mass Spectrometry, Institute of Analytical Chemistry, University of Leipzig, Leipzig, Germany; 5 Institute of Physiological Chemistry, Faculty of Veterinary Medicine, University of Leipzig, Leipzig, Germany; 6 German Centre for Integrative Biodiversity Research (iDiv), Leipzig, Germany; University of California-Davis, UNITED STATES

## Abstract

Previous studies showed that olfactory cues are important for mammalian communication. However, many specific compounds that convey information between conspecifics are still unknown. To understand mechanisms and functions of olfactory cues, olfactory signals such as volatile compounds emitted from individuals need to be assessed. Sampling of animals with and without scent glands was typically conducted using cotton swabs rubbed over the skin or fur and analysed by gas chromatography-mass spectrometry (GC-MS). However, this method has various drawbacks, including a high level of contaminations. Thus, we adapted two methods of volatile sampling from other research fields and compared them to sampling with cotton swabs. To do so we assessed the body odor of common marmosets (*Callithrix jacchus*) using cotton swabs, thermal desorption (TD) tubes and, alternatively, a mobile GC-MS device containing a thermal desorption trap. Overall, TD tubes comprised most compounds (N = 113), with half of those compounds being volatile (N = 52). The mobile GC-MS captured the fewest compounds (N = 35), of which all were volatile. Cotton swabs contained an intermediate number of compounds (N = 55), but very few volatiles (N = 10). Almost all compounds found with the mobile GC-MS were also captured with TD tubes (94%). Hence, we recommend TD tubes for *state of the art* sampling of body odor of mammals or other vertebrates, particularly for field studies, as they can be easily transported, stored and analysed with high performance instruments in the lab. Nevertheless, cotton swabs capture compounds which still may contribute to the body odor, e.g. after bacterial fermentation, while profiles from mobile GC-MS include only the most abundant volatiles of the body odor.

## Introduction

The olfactory sense is a vital channel for mammalian communication and is part of chemosensing, which is the phylogenetically oldest sensory system [1]. Individuals can use body odor to discriminate individuals [2] and recognize the sex [3] as well as kinship [4] of conspecifics. Moreover, behavioral and physiological characteristics such as the dominance rank and menstrual cycle states [5] are conceivable from body odor. Still, olfaction is considerably less studied in mammals compared to visual or auditory cues, presumably caused by methodological difficulties in measuring olfactory properties [6].

Olfactory communication operates via volatile as well as semi- or nonvolatile compounds from different sources such as specialized scent gland secretions, sweat, urine or feces [7]. In particular, body odor comprises also volatiles which are formed from nonvolatiles by metabolic degradation of skin bacteria [8]. Volatile scent compounds are directly detectable by the main olfactory epithelium [9] of a receiver and thus are of particular interest when studying body odor. Commonly, cleaned cotton swabs are rubbed over the skin or fur of mammals to capture body odor substances [7]. This method is well established and affordable, but samples need to be stored immediately at– 80°C [10] and animals need to be trained for the direct contact. In previous studies that used cotton swab samples of scent glands, the samples contained more semi- or nonvolatile compounds than volatiles. For instance, none of 32 compounds using liquid extraction [11] and 21 of 42 compounds using headspace extraction were volatile [12]. Also, a recent investigation of body odor samples of rhesus macaques (*Macaca mulatta*) taken with cotton swabs revealed semi-volatile rather than volatile compounds and many contaminations from sampling and materials of the liquid extraction in addition to the substances of interest [13]. Thus, a method for collecting volatiles that allows for a low-loss and easy storage of samples and introduces less contaminations would be highly desirable. Hence, we searched for established methods used in other fields which could be applicable to body odor sampling in mammals. Various methods of volatile sampling are employed, however, comparisons on odor sampling techniques were often drawn for investigations on plants, but rarely for mammals [14].

Plant and environmental studies commonly use adsorbent materials which are, for instance, packed in thermal desorption (TD) tubes [15–19]. These materials adsorb substances present in the air and retain them in closed TD tubes even under ambient temperature. Furthermore, a direct animal contact is not needed, because the air near to the skin is sampled. In a first study adapting TD tube sampling to a mammalian species, sampling flow rate and volume could be successfully adjusted to a feasible sampling duration of 20–40 s per sample (Weiß et al. under revision). Additionally, the type of adsorbent material can be chosen dependent on characteristics of interaction with the substances of interest given by their volatility or polarity [20]. To capture a broader range of substances, also multiple sorbents can be combined [21]. Hence, the usage of TD tubes provides flexible options and could be a promising alternative to the sampling of body odor with cotton swabs.

Mobile GC-MS analysis is another option for sampling and analyzing volatiles. For example, the mobile GC-MS HAPSITE^®^ ER was originally developed for volatile analysis within the context of emergency preparedness and hazard detection. It contains a system-inherent TD trap and analyses the samples immediately after sampling by suction with a probe. This device could be suitable for sampling mammalian body odor even in the field and does not require any sample storage. Sample analysis with this mobile GC-MS is considerably shorter compared to laboratory instruments. Thus, the quality of the analytical performance of the mobile device would be a key property to decide whether it is an appropriate alternative method for sampling body odor.

Consequently, we conducted a comparison of chemical profiles based on body odor of animal samples collected in parallel with three methods. We contrasted cotton swabs commonly used for sampling animals with two methods routinely used in different research fields and newly applied for mammalian body odor: TD tubes and mobile GC-MS. As a first check for the suitability of the approaches in general, we assessed the overall number of captured chemical compounds and the proportion of volatiles therein. In particular, we regarded the method providing access to the highest number of volatiles and an analysis with high sensitivity and resolution as most suitable for detecting and identifying compounds used for communication. Furthermore, the sets of captured volatiles were compared between the methods to determine whether complementary results were produced, meaning that we would find completely unique sets of compounds, or whether one method outperforms others by covering an already detected set of compounds, still having a varying number of unique compounds per method in addition to that. Finally, two different single adsorbent materials and a combination of two adsorbents were evaluated for sample collection with TD tubes. This would allow to determine whether compounds are specifically captured with one material and whether a combination of two adsorbent materials is beneficial. Our aim was to infer the most comprehensive method for body odor sampling to study olfactory communication in mammals. We further discuss the ease of application of all three methods to provide support for the choice of the most appropriate method for future studies.

## Materials and methods

### Sampling materials

We used various sampling methods in parallel to determine the best performance for capturing volatile organic compounds (VOCs) in mammalian body odor. First, we used cotton swabs (Lilibe Cosmetics, Rossmann) composed of 60% cotton wool, 20% micro fiber from polyester and 15% polyester which were cleaned prior use by baking for 30 min at 130°C [13]. Second, we applied thermal desorption tubes containing various adsorbent materials with specific affinities for different substances regarding volatility and polarity [22,23]. We tested TD tubes containing either Tenax TA (60/80 mesh, stainless steel TD tube, 1/4 in. × 31/2 in., Sigma-Aldrich, Steinheim, Germany) or XAD-4 (160/80 mg with glass fiber filter, 13 to 8 mm × 75 mm ORBO 65P, OVS fluoride tube, Sigma-Aldrich, Steinheim, Germany). Furthermore, we used TD tubes with two materials (hereafter “TD tubes Mix”: stainless steel TD tube, 1/4 in. × 3 1/2 in., 0.095 g of Tenax TA, 0.2104 g of XAD-2), separated by 2 mm of glass wool (Supelco, Bellefont, USA) which were closed with Swagelok Brass caps (1/4 in., Supelco, Bellefont, USA). The two amberlite adsorbents XAD-2 and XAD-4 are very similar in regard to compound adsorption [24]. Prior to the sampling, TD tubes containing the adsorbent combination were cleaned by baking under nitrogen flow (50 mL/min) for 152 min in a TD Clean Cube device (SIM Scientific Instruments Manufacturer GmbH, Oberhausen, Germany) starting at 40°C and increased up to 200°C. For sampling, TD tubes were connected to a pump (BiVOC2, Umweltanalytik Holbach GmbH, Germany). Third, we assessed a mobile GC-MS device (HAPSITE^®^ ER, Inficon Inc., East Syracuse, NY, USA) containing a TD trap with three proprietary adsorbent materials (TriBed microconcentrator).

### Animals and sampling

For each method, two samples per individual were taken from the genital area of six female common marmosets (*Callithrix jacchus)* leading to 12 samples per method (except of XAD-4 with 10 samples) and a total of 58 samples. The animals belonged to a colony of the Faculty of Veterinary Medicine of the University of Leipzig (Germany). They were housed in pairs and trained for a regular handling [25]. All cotton, mobile GC-MS and TD tube Mix samples were collected in parallel per subject across 10 days in 2015. In addition, TD tube Tenax TA and XAD-4 samples were taken on only 3 of the 10 d (see S1 Table). If animals were previously sampled two times in parallel with the three sampling methods, TD tubes Tenax and XAD-4 samples were taken on separate days without resampling with the other methods for animal welfare. Those samples were parallel in regard to the animals ID, but not to the sampling date (Tenax TA: 7, XAD-4: 6). If parallel samples of the three sampling methods had still to be sampled, TD tubes Tenax TA and XAD-4 samples could be taken in parallel from the same animals and at the same day (Tenax TA: 5, XAD-4: 4). The regional board of Leipzig, Germany approved animal husbandry (reference number: 24-9168.11/17/68) and research was authorized by the Regional Council Leipzig, Germany (TVV 16/15). Body odor sampling is noninvasive and needs no authorization under the German Animal Welfare Act. However, the study was in accordance with the German Animal Welfare Act for the care and use of laboratory animals.

Cotton swabs were rubbed over the skin of the animal for 20 s. Immediately after sampling, the swabs were transferred into precleaned glass vials (4 mL, Carl Roth GmbH & Co. KG, Karlsruhe, Germany) and stored at– 80°C until analysis. The sampling duration for TD tubes and mobile GC-MS were intended to not exceed four min. Sampling with the mobile GC-MS (300 mL) lasted three min, whereas the sampling durations of TD tubes varied with the adsorbent types. Under field conditions, the maximum flow would be preferred to decrease the sampling duration as much as possible. Hence, we customized the sampling parameters to get a comparable setting with constant durations as the critical sampling parameter. Due to variation in maximal flow rates (TD tubes Mix and XAD-4: 1.1–1.5 L/min; TD tubes Tenax TA: 0.4–0.6 L/min), the sample volumes needed an adjustment per method (TD tubes Mix and XAD-4: 6 L; TD tubes Tenax TA: 2 L) to result in comparable sampling durations. Air from near to the skin (approx. 1 cm) was collected. TD tubes were stored at room temperature until analysis. Mobile GC-MS samples were analysed immediately after sampling.

### GC-MS analysis

Samples of different sampling methods and adsorbent types were measured on different instruments to ensure optimal analyses of the respective sample types. The chemical compounds from cotton swab samples were extracted with 1.2 mL of n-hexane (Sigma Aldrich, Steinheim, Germany) and stepwise concentrated to 60 μL by evaporation. For GC-MS analysis, 4 μL of this solution was injected at 250°C using splitless injection during 2 min into an HP6890 Series GC System with a Mass Selective Detector HP5973 MSD (Agilent, Waldbronn, Germany) in electron-impact ionization mode at 70 eV. The scan range was set to *m/z* 50–550. GC separation was accomplished on a DB35-MS column from J&W Fisher (30 m length, 0.25 mm inner diameter (ID), 0.25 μm film; Agilent, Waldbronn, Germany) and Helium 5.0 (alphagaz, Air Liquide, Düsseldorf, Germany) was used as carrier gas with a linear velocity of 46.8 cm/s (flow rate 1.7 mL/min). The temperature program started at 35°C held for 2 min, followed by a 10°C/min temperature increase until 320°C, held for 10 min.

The TD tube Mix samples were analysed using a GCMS-TQ8040 with a Gas Chromatograph GC-2010 Plus and a Triple Quadrupole Mass Spectrometer coupled to a thermal desorption system TD-20 (Shimadzu, Kyoto, Japan) for introducing the samples. Injection was done in split mode (split ratio 5), samples were desorbed for 8 min at 250°C with a helium flow of 60 mL/min to a Tenax TA-filled cold trap (-20°C). The cold trap was heated up to 250°C under a helium flow of 14.5 mL/min and desorbed into the gas chromatograph through the opened outlet split at 140.2 kPa. Electron-impact ionization (EI) was carried out at 70 eV and 200°C and the scan range was set to *m/z* 30–300. The GC was equipped with two columns connected to each other (Rxi-1ms: 30 m length, 0.25 mm ID, 0.25 μm film, and SGE Analytical Science BPX50: 2 m length, 0.15 mm ID, 0.15 μm film, Restek GmbH, Bad Homburg vor der Höhe, Germany) and used for one dimensional chromatography. Helium 5.0 was used as carrier gas with a linear velocity of 35 cm/s (flow rate 1.58 mL/min). The temperature program started at 35°C for 0.5 min, followed by a temperature gradient of 6°C/min until 320°C, held for 25 min.

Commonly, due to its lower thermal stability [26] XAD resins are used to adsorb compounds from solvents followed by liquid extraction. For this reason, liquid extraction was used for the TD tubes containing only XAD-4. Chemical compounds captured with TD tubes XAD-4 were extracted with 0.5 mL of a methanol/dichloromethane solution (1:1) in an ultrasonic bath. After that, 50 μL of this solution was introduced to 2D-GC separation on an Agilent GC 7890A using a Gerstel KAS4 large volume injection device. The GC contained two columns: Agilent VF 5ms (first dimension, 30 m length, 0.25 mm ID, 0.25 μm film) and Restek RTX-200 (second dimension, 3 m length, 0.18 mm ID, 0.18 μm film). The carrier gas was helium 5.0 with a linear velocity of 26 cm/s (flow rate 1 mL/min), cryofocussing before the second dimension was accomplished using liquid nitrogen during a modulation time of 5 s followed by a hot pulse of 1 s duration. The temperature program started at 40°C for 1 min followed by heating with 5°C/min first to 110°C held for 0.01 min, then to 220°C held for 0.01 min and finally to 280°C, held for 6 min. The secondary oven had an offset of + 10°C. The mass scan range was *m/z* 30–300 with a 200 spectra per min scan rate in the TOF detector of a Pegasus 4D GCxGC TOFMS device (Leco Corp., Saint Joseph, MI, USA).

TD tubes containing Tenax TA were measured on the same GC-MS system Agilent 7890 GC/Pegasus 4D GCxGC TOFMS (Leco Corp., Saint Joseph, MI, USA) this time coupled with a thermal desorption injector T-ATD (PerkinElmer Inc., Waltham, MA, USA). Samples were purged for 1 min and afterwards desorbed at closed inlet split for 3 min at 260°C with a helium flow of 100 mL/min to a Tenax TA filled cold trap (- 30°C). The cold trap was heated up to 270°C under a helium flow of 10 mL/min and injected into the gas chromatograph through the opened outlet split at 170 kPa. Helium was the carrier gas at a linear velocity of 26 cm/s (flow rate 1 mL/min). The temperature program started at 40°C for 8 min, followed by heating steps of always 5°C/min until 160°C held for 0.6 s, afterwards heated up to 250°C, held for 0.6 s and lastly to 275°C, held for 12 min and the secondary oven had an offset of + 10°C. The mass scan range was set to *m/z* 30–300 with 200 spectra per min.

The mobile GC-MS HAPSITE^®^ ER (Inficon Inc., East Syracuse, NY, USA) featured a polydimethylsiloxane column (15 m length, 0.25 mm ID, 1.0μm film) and using ultra high purity nitrogen as carrier gas with a linear velocity of 74.4 cm/s (flow rate 2 mL/min). The ionization mode was EI at 70 eV and the mass scan range was set to *m/z* 41–300. The temperature program started at 60°C held for 1 min, followed by heating with 60°C/min to 80°C, 12°C/min to 120°C and 26°C/min to a final temperature of 180°C.

### Data analysis

#### 1. Compound coverage: Number and volatility of target substances detected by the different approaches

For the overall comparison of the total number of peaks and their volatility, we determined those peaks per sampling method (cotton swabs, TD tubes Mix and mobile GC-MS) which occurred per method in at least half of the samples. In the chromatograms obtained from the cotton swabs and the TD tubes Mix, peak picking was done with AMDIS v. 2.65 [27]. For the samples analysed by the mobile GC-MS, peak picking was accomplished with the software ER IQ (Inficon Inc., East Syracuse, NY, USA), respectively. For all three sampling approaches, repeatedly occurring peaks within a narrow span of retention times for a given analysis set were defined as “RT range” and correspond to the number of compounds detected with a particular method. The animal sample RT ranges were compared to the corresponding RT ranges from blank samples to identify those compounds originating most likely from the marmosets and not from the environment or the sampling material. Only RT ranges with higher areas in the animal samples than in the blank samples were considered for further evaluation. Additionally, tentative substance identifications were assigned to these compounds using the NIST14 Mass Spectral Library (National Institute of Standards and Technologies, Gaithersburg, MD, USA). Only library hits which were suggested for a particular RT range in the majority of all samples with appropriate matches (> 700) were considered. In addition, some compounds were identified by co-spiking (parallel measurement of authentic standards to animal samples, see S2 Table). Finally, the boiling points of the compounds obtained from ChemSpider ([28], predicted values from ACD/Labs Percepta Predictors) were used to define a threshold between volatile (< 250°C) and semi-volatile (> 250°C) compounds [29]. As only a minority of cotton swab compounds was volatile, the cotton swab samples could not be incorporated into the further comparison of volatiles between the sampling methods.

#### 2. Overlap of volatiles in thermal desorption tubes and mobile GC-MS samples

Using the automated peak integration of GCMS solution v. 4.20 (Shimadzu, Kyoto, Japan), all TD tube Mix peaks were integrated and the relative area was determined as the peak area divided by sum of all peak areas in the respective chromatogram (i.e. “peak sum”). We used the elution order and two specific *m/z* (mass to charge ratio, equal to mass within the mass spectra for GC-MS) to check which of the compounds detected by the mobile GC-MS were also detected with the TD tube Mix. To assess if the detection of a compound by one or both methods was related to the abundance of the respective compound, we conducted a linear mixed model [30] in R version 2.3.2 (R Core Team 2015) using the function lmer of the R-package lme4 [31]. The model compared relative peak areas of the TD tube Mix compounds which were in common with the mobile GC-MS to the relative peak areas of unique TD compounds (absent in mobile GC-MS samples). Relative peak area was set as response, the sample set (“common” or “TD unique”) was the predictor and sample number as well as identity of the animal were incorporated as random effects. Additionally, we included random slopes of sample set within sample number and identity, but left out the correlation between random intercepts and random slopes for convergence reasons [32,33]. Prior to fitting the model, we checked the response for data distribution and log-transformed the relative peak areas, resulting in a slightly right-skewed, but normal distribution. We inspected a qqplot and a scatterplot of the residuals plotted against fitted values and found no serious deviations from the assumptions of normally distributed and homogenous residuals. Furthermore, we checked the model stability, which provided no indication for influential cases. A likelihood ratio test using the function anova was done to examine the significance of the full model in comparison to the null model lacking the random effect sample set [34,35].

#### 3. The impact of sorbent type on VOC analysis

Additionally to the TD tube Mix samples (N = 12), we sampled body odor of our test animals with TD tubes containing either only Tenax TA (N = 12) or only XAD-4 (N = 10) analysed by 2D GC-MS as described above. For peak picking and substance identification, the software Chroma-TOF 4.51.6.0 (Leco Corp, Saint Joseph, MI, USA) and NIST14 was used. Tentative substance identifications were used to identify compounds which were in common to two or all types of adsorbents. To confirm this assignment we first correlated the elution order of these compounds between the adsorbent types using Spearman rank correlations in R version 2.3.2 [36]. To assess how similar the recovery patterns (relative intensities) of these compounds were for the different adsorbent types, we also subjected the mean relative areas of the compounds between adsorbent types to a Spearman rank correlation analysis.

#### 4. Confirmed and most abundant compounds of all methods

Finally, we wanted to provide a deeper insight into the type of compounds present in the samples of common marmosets taken via all three methods and adsorbent types. Therefore, we assembled a list of compounds with confirmed identity (through peak identification utilizing authentic standards which we applied only for samples of TD tube Tenax TA, TD tube XAD-4, TD tube Mix) together with the most abundant compounds detected from cotton swabs, TD tubes Tenax TA, XAD-4 and Mix as well as mobile GC-MS sampling. Based on a literature research, we classified these compounds into categories of their origin. Compounds labelled as “exogenous” are most likely not from the animals, as they are known to be contaminants and hence presumably originate from the environment during sampling, the sampling material or chemicals from the laboratory during processing. Compounds labelled as “potentially endogenous” have been described to originate from animals in previous studies and we found no hint of an exogenous source. Given the potential to trap compounds which could have been metabolized by the animals or skin bacteria, even if originally not produced by the animals, we introduced the classification “potentially metabolized”. This category applies to all substances which have been reported for nonnatural origin (see above “exogenous”) as well as for animal origin (“endogenous”) and cannot be assigned to one of both. Metabolized compounds can also be informative about the target animal, because metabolic processes may considerably vary with e.g. the age, sex, group membership or reproductive state of the host [37–39]. Some compound identifications could not be found in the literature and thus could not be associated with one of the categories and remain as “unclassified”.

## Results

Prior to blank correction, we detected total numbers of unique peaks of 249 for cotton swabs, 41 for the mobile GC-MS, 375 for TD tubes Mix, 308 for TD tubes Tenax TA and 265 for TD tubes XAD-4. For a quantitative evaluation, we considered a set of 109 manually curated body odor compounds sampled with the tested sampling methods. This set is composed of 49 compounds for which identity was confirmed by co-spiking with authentic standards and 60 of the most abundant compounds which were tentatively identified by library search (see S2 Table).

The investigation of boiling points revealed 77% volatile compounds (bp < 250°C) and 23% semi- or nonvolatiles in the set of 109 compounds. We classified all compounds into categories of their origin and found that 34% of them are potentially endogenous, 50% are potentially metabolized and only 12% are exogenous, as well as 4% unclassified compounds. Exogenous compounds were, for instance, plasticizer (for example 2-butanedioic acid (*Z*)-, diethyl ester) and solvents (for example triethylamine). Many of the potentially metabolized compounds were alkanes (for example hexadecane), which are most likely metabolic products of skin bacteria [40], or compounds known to originate from plants (for example β-ionone) which could result from food metabolized by the animals. Compounds probably originating from the animals themselves were mainly alcohols, carboxylic acids and their esters, as well as aldehydes and ketones (see S2 Table), very well depicting the skin volatiles reported in the literature [41]. Thus, most of these compounds (84%, composed of potentially endogenous and metabolized substances) could be biologically relevant odor compounds. However, the ratios of volatility as well as origin varied between the sampling methods. Within the 109 compounds considered in our study, the highest number of potentially biologically relevant compounds were found for TD tubes Tenax TA (97%) and Mix (90%) indicating a lower degree of exogenous compounds (contaminations).

### 1. Compound coverage: Number and volatility of target substances detected by the different approaches

The three sampling methods, cotton swabs, TD tubes and mobile GC-MS, captured different numbers of compounds from animal samples (Table 1).

**Table 1:** Overview about the number of detected compounds.

	Cotton	Mobile GC-MS	TD tube
**Compounds in total**	55	35	113
**Compounds volatile**	10 (18%)	35 (100%)	52 (46%)

Total number of detected compounds as well as number and percentage (in brackets) of volatile compounds of animal origin (not present in higher amount in blanks) from TD tubes Mix (TD tube), cotton swabs (Cotton) and the mobile GC-MS.

Among these different methodologies, TD tube samples contained the most compounds likely originating from the animals (90%) and the mobile GC-MS and cotton swab samples the fewest (72% and 73%). In addition, from the total amount of compounds only a minor part was volatile for cotton swabs, but half of the TD tube Mix and all of the mobile GC-MS samples were volatile (see Table 1 and example chromatograms in Supplementary, S1 Fig). Overall, the TD tubes comprised the highest total number of substances and the highest number of volatile and substances of animal origin.

### 2. Overlap of volatiles in thermal desorption tubes and mobile GC-MS samples

As the mobile GC-MS appeared to be less sensitive (fewer substances were detected), we expected that most of the compounds detected with the mobile GC-MS would be present in the larger set of compounds found in the TD tube Mix approach. Indeed, 33 of 35 (94%) volatile compounds detected with the mobile GC-MS were also captured with TD tubes Mix and only two unknown (without substance identification) compounds were unique for the mobile GC-MS.

Of the 33 volatile compounds in common, 18 could be tentatively identified by library comparison, which comprised 13 potentially endogenous or potentially metabolized and only five exogenous compounds (see Table 2). From the compounds in the TD tube chromatograms (total N = 113), 27 were identified by co-spiking and an additional 15 were annotated by library search (see S2 Table).

**Table 2:** Compounds in common to TD tubes Mix and mobile GC-MS samples.

		Mobile GC-MS			TD tubes Mix		Classification	
Substance	Bp°C	RT	Area	Mt	RT	Area	Origin	Ref
1-Propanol	95±3	1.00	320,802,296	937	2.78	86,417,340	pot endo	[42,43]
Cyclohexane, methyl-	101±3	2.06	3,895,100	715	4.54	31,159,370	pot metab	[44,45]*
Toluene	111±3	2.29	2,896,431	838	5.11	2,264,769	exo	[46]
Heptane, 2-methyl-	118±3	2.35	851,165	575	4.97	111,756,372	pot metab	[43]
Hexanal	128±3	2.45	5,600,184	735	5.38	2,905,761	pot endo	[47,48]*
Propanoic acid, propylester	122±3	2.57	1,922,248	745	5.91	272,145	pot endo	[42]
Ethylbenzene	136±3	3.54	4,087,496	839	7.13	342,180	exo	[43,46]*
Pyrazine, 2,5dimethyl-	155	4.38	71,719,045	854	8.05	75,802	pot metab	[43,49]
Nonane	152±3	4.49	980,197	606	8.40	275,808	pot metab	[46,50]*
Benzene, (1methylethyl)-	152	5.02	1,043,167	687	8.69	6,813	exo	[26]
Benzaldehyde	179	5.27	62,440,110	900	9.16	271,986	pot metab	[46,49]*
5-Hepten-2-one, 6-methyl-	173±9	6.03	5,492,894	644	10.03	254,562	pot metab	[46,48]*
D-Limonene	175±20	7.04	44,230,737	887	11.50	107,289	pot metab	[43]
Acetophenone	202	7.19	11,704,738	667	12.18	193,246	exo	[42,51]*
Nonanal	191±3	8.00	114,615,201	823	13.92	9,186,924	pot metab	[46,47]*
Undecane	197±3	8.15	55 673,854	742	14.49	18,506,217	exo	[12,48]*
Decanal	209±3	9.17	11,160,309	790	15.63	5,270,498	pot endo	[45,46]*
Dodecane	216±3	9.24	8,432,826	716	15.99	2,401,000	pot endo	[47,48]*

Tentative substance identifications of common compounds including the boiling point (bp, in °C at 760 mmHg, ChemSpider [28], retention time (RT, in min), mean area (Area), mean match (Mt) of substance suggestion, presumable origin (Origin: exo = exogenous, pot endo = potentially endogenous, pot metab = potentially metabolized) as well as references (Ref) of other body odor studies reporting the respective substance (* = more references see S2 Table).

The high correlation of the retention times in the samples from TD tubes Mix and the compounds detected with the mobile GC-MS supported the correct assignment of the compounds in common (Spearman rank correlation: N = 17, r = 1, p < 0.001). In contrast, the relative mean areas of shared compounds were not significantly correlated (Spearman rank correlation: N = 17, r = 0.31, p = 0.168). Hence, the response patterns of volatiles differed between the two sampling methods.

**Fig 1:**
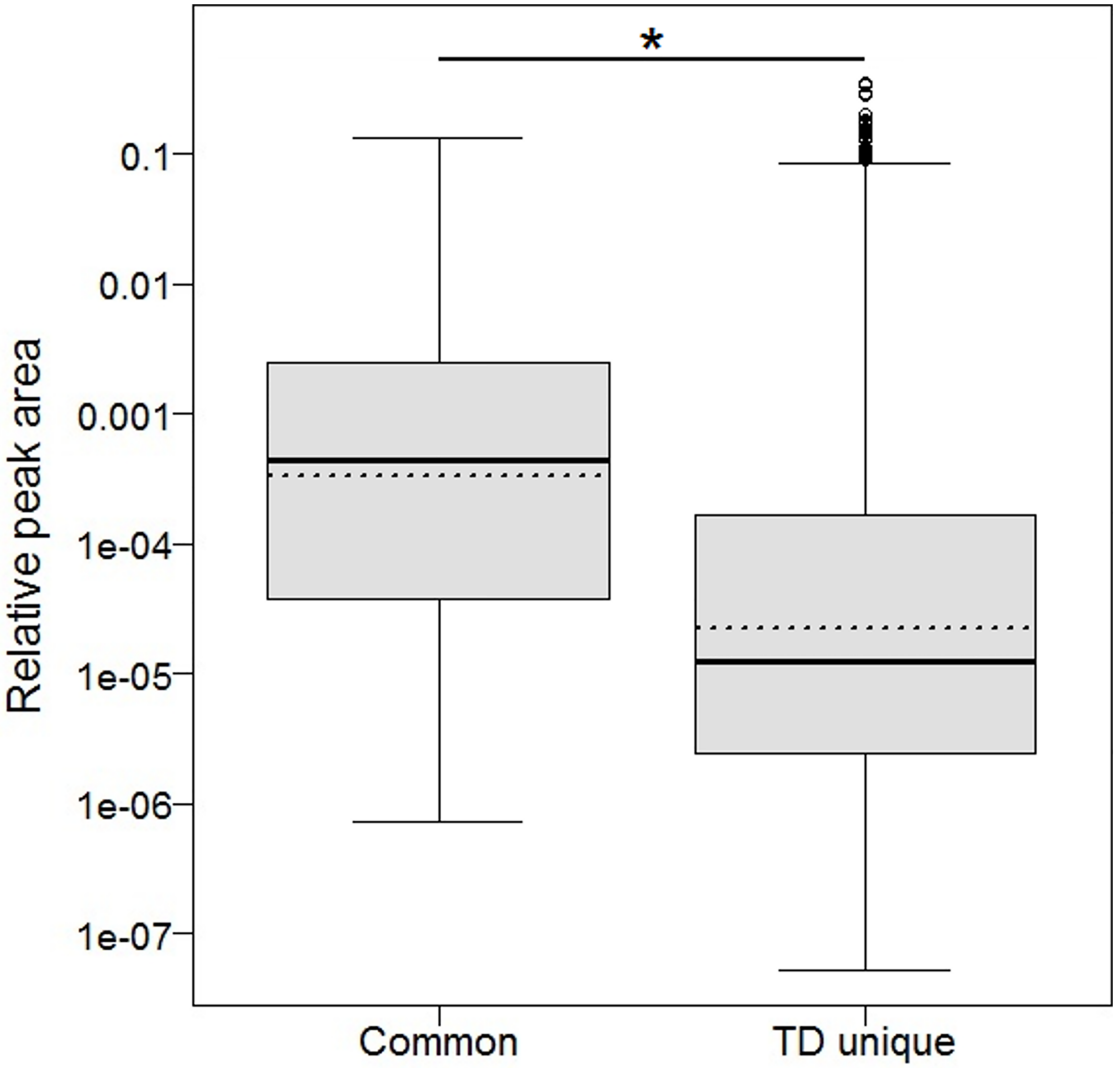
Comparison of TD tube peaks which are in common with the mobile GC-MS and occurring only in TD. Box-and-whisker plots of the relative peak areas from integration of TD tubes Mix peaks in common with the mobile GC-MS samples (left, Median_common_ = 4.25 x 10^-4^, N_common_ = 183) and peaks which are unique for TD tubes Mix (right, Median_TD_ = 0.12 x 10^−4^, N_TD_ = 9217). Bold, solid lines indicate medians, boxes range from the lower to the upper quartiles, lower whiskers are defined as max(min(x), Q_1–1.5 * IQR), upper whiskers equal min(max(x), Q_3 + 1.5 * IQR), open circles are outliers, dotted lines depict the means and asterisk indicates the significant difference. The relative peak areas were log transformed, the y-axis of the plot is on original scale.

Finally, relative peak areas of compounds from TD tubes Mix which were also found with the mobile GC-MS were significantly higher than of the compounds detected only in TD tubes Mix (Fig 1, linear mixed model: χ2 = 23.35, df = 1, p < 0.001). In other words, the mobile GC-MS detected the most abundant of the compounds found in TD tubes Mix as well.

### 3. The impact of sorbent type on VOC analysis

To ascertain whether the combination of two adsorbent materials resulted in more captured compounds than single adsorbents, we specifically searched for the 27 volatile compounds whose identification was confirmed by co-spiking authentic standards (see also S2 Table). Of the 27 confirmed compounds, 22 were identified by co-spiking in Tenax TA and six in XAD-4 liquid desorption. Only 14% of the 22 compounds confirmed for Tenax TA were retrieved with XAD-4 and also only 33% of the six compounds confirmed for XAD-4 were retrieved with Tenax TA (see Table 3). However, TD tubes Mix, which contained two adsorbent types, retrieved almost all of the volatiles detected with Tenax TA alone (91%) as well as all compounds from XAD-4. Hence, the combination of adsorbents indeed captured more compounds than when using a single adsorbent.

**Table 3:** Number of confirmed compounds retrieved dependent on the adsorbent used.

27 authentic standards	Retrieved in Tenax TA	Retrieved in XAD-4	Retrieved in Mix
22 Tenax TA standards	22 (100%)	3 (14%)	20 (91%)
			Corr RT: ρ = 0.86, p < 0.001
			Corr Area: ρ = 0.06, p = 0.776
6 XAD-2 standards	2 (33%)	6 (100%)	6 (100%)
			Corr RT: ρ = 0.94, p = 0.016
			Corr Area: ρ = -0.31, p = 0.564

Compounds confirmed in Tenax TA (after thermal desorption) and XAD-4 (after liquid extraction) were searched in samples of the other adsorbent and the mixture of two adsorbents (TD tubes Mix). Number and percentage of retrieved compounds are listed along with correlations of retention times (Corr RT) and relative mean areas (Corr Area) between retrieved (TD tubes Mix) and confirmed (Tenax TA as well as XAD-4) compounds.

Finally, to check whether the recovery patterns of compounds found with the different adsorbent materials are similar, we correlated the relative mean areas of compounds retrieved with TD tubes Mix with both TD tubes Tenax TA and TD tubes XAD-4. Beforehand, we evaluated the accurate assignment of shared compounds by correlating their retention times. In agreement with a correct assignment, the retention times of the compounds analysed from TD tubes Mix highly correlated with both Tenax TA and XAD-4 (Spearman rank correlation, Tenax TA: N = 20, r = 0.86, p < 0.001; XAD-4: N = 6, r = 0.94, p = 0.016). In contrast, correlations of relative mean areas revealed no significant relationship between the methods at all (Spearman rank correlation: Tenax TA: N = 20, r = 0.06, p = 0.776; XAD-4: N = 6, r = -0.31, p = 0.564). Thus, compounds exhibited a completely different response pattern with the different adsorbent materials and combinations. In particular, two compounds which were exceedingly more abundant in TD Mix tubes than expected by comparison to Tenax TA were both ketones and two compounds which were recovered with a remarkably lower intensity in TD Mix tubes were both alcohols (data not shown).

## Discussion

We compared three different sampling methods for subsequent GC-MS analysis of mammalian body odor, including cotton swab sampling, sampling with thermal desorption tubes, and by direct probe with a mobile GC-MS. Due to their ease of transmission, volatile compounds are most interesting when studying the function of body odor for olfactory communication. The methodological difficulties are to reproducibly capture these volatiles, transfer them into the GC-MS for analysis and to get sufficient quality to differentiate compounds originating from the study animals from contaminants within the chromatogram. We found considerable differences in the performance of the methods, whereby volatile compounds could be sampled with two out of the three methods.

### Cotton swabs

Sampling with cotton swabs has been the most commonly used method in studies of mammalian odor [7]. However, we found that cotton swabs capture considerably fewer volatile compounds than the other two methods and very few volatiles at all. Probably, rubbing a swab over the skin of an animal provides insufficient interaction and retention for a satisfactory adsorption of volatiles to the cotton swab [13]. Hence, our study confirms that this method is unsuitable for detecting the volatile compounds directly available for olfactory communication. However, also the semi- and nonvolatiles captured by cotton swabs can play a role in communication. Bacteria present on the skin of mammals can metabolize compounds into volatiles [52]. According to this fermentation hypothesis, semi- and nonvolatiles can thus contribute indirectly to olfactory communication [37–39]. Furthermore, mammals possessing a vomeronasal organ may also be able to directly perceive semi- and nonvolatile compounds to some extent [53]. Thus, cotton swabs capture a particular part of the animal’s chemical profile, but not the volatiles of the body.

Another critical aspect of the reduced suitability of cotton swabs is the high background in the chromatograms. Many contaminations originate from the cotton material itself or the solvents used for extraction [13] and need first to be identified as exogenous and excluded from further analysis. In the present study, many of the most abundant substances detected in the cotton swabs were surfactants belonging to the background or alkanes which could be exogenous or metabolic products from bacteria on the skin of the animals [40]. Unique for cotton swabs were steroids (for example cholesterol and its esters) which are most likely of endogenous origin [54], although they are not volatile. To alleviate the problem of contaminations, a suitable material other than cotton, which is itself a biological material, could be selected for swab sampling, such as viscose-type swabs [13].

### Mobile GC-MS

The mobile GC-MS, originally developed for application in hazard detection [55], exclusively captured volatile compounds. The compounds for which we obtained acceptable library identifications mainly covered the range of skin volatiles previously reported in humans and nonhuman primates, for instance alkanes, aldehydes, short-chain alcohols, esters of carboxylic acids and ketones [41] as well as aromatic hydrocarbons [54]. The total number of volatile compounds, however, was clearly lower than for the TD tubes Mix (Hapsite: 35, TD tubes Mix: 52). An alignment of the volatiles captured with the two methods revealed that almost all compounds detected with mobile GC-MS were detected as well with TD tubes Mix. Thus, the mobile GC-MS is capable of sampling volatile body odor compounds, but the detected compound set is smaller and biased towards the most abundant compounds captured with the TD tubes Mix. Furthermore, the relative areas of compounds found with both, the mobile GC-MS and the TD tubes Mix, were not correlated and consequently, compounds are variously depicted by the two methods. This could be due to the different adsorbent materials or the different GC-MS devices used for analysis. Additionally, owing to the short GC-MS analysis, the resolution of the mobile device was clearly outcompeted by that of a laboratory device (data not shown). Moreover, in the mobile device used in this study, the chromatographic data format is highly specific, limiting data exchange and chromatogram evaluation to the manufacturer’s software. These aspects considerably impeded compound identification even though the background noise was low. Hence, although a mobile GC-MS would allow data analysis without prior storage of samples directly in the field, its lower sensitivity and more difficult data analysis than with lab methods make pilot studies a prerequisite for assessing its applicability to a particular species and question. In particular, we consider its use feasible only for studying olfactory communication if compounds of interest were identified in prior studies using a more sensitive sampling method and if the detectability of these compounds of interest with a mobile GC-MS was verified in pilot studies.

### TD tubes

Thermal desorption tubes, commonly used for dynamic head space sampling in plant studies [56], comprised most compounds in total and more volatiles compared to the other two methods. TD tubes Mix contained many compounds which were not captured by the mobile device, whereas almost all mobile GC-MS compounds were also detected in TD tube Mix samples. In comparison to cottons swabs, containing almost only semi- and nonvolatiles, and the mobile GC-MS with exclusively volatile compounds, TD tubes cover the broadest range of volatility. Even if none of the methods is able to reflect all aspects of chemical profiles, TD tubes are the most convenient method to sample body odor. Another important advantage of TD tubes is the possibility to choose adsorbent materials, which can also be combined in one tube, dependent on the compounds of interest.

### Various adsorbent types

Similar to the mobile GC-MS, confirmed and most abundant compounds of the TD tubes with all three different adsorbent materials detected skin volatiles already described previously [41] including short-chain alcohols, aldehydes and ketones as well as carboxylic acids and their esters. However, the recovery patterns of the three different TD tubes Tenax TA, XAD-4 and TD tubes Mix are markedly different. This lack of a correlation of relative areas could be driven by various factors. Each adsorbent type is specific for particular compound classes and thus, same compounds are conveyed in different amounts using different adsorbent types. For instance, in the comparison between TD tubes Tenax and Mix, two ketones and two alcohols were recovered with particularly distinct, deviating amounts. The combination of multiple adsorbents resulted in different patterns as well, because some substances are captured by both and some only by one of the materials. In addition, the sampling of Tenax TA und XAD-4 was not totally parallel in time to the samples of TD tubes Mix. This could probably result in further variation of compound detection, although the same methodology (nonpolar GC coupled to standard EI mass spectrometry) was employed and the correlation of the retention times supported the correct annotation of the compounds in the analysis with the different GC-MS instruments. The deviating use of a different instrumental device for Tenax TA and XAD-4 as well as the different desorption protocol of XAD-4 in comparison to TD tubes Tenax and Mix might contribute to the observed variance of recovery patterns in our samples, too.

We found that several compounds which were confirmed for either Tenax TA after thermal desorption or XAD-4 after liquid extraction were only partly detectable using the other adsorbent material. This discrepancy could be to some extend ascribed to the different desorption protocol employed in our investigation for the two materials. In particular, liquid extraction of XAD-4, which is much more cumbersome than thermal desorption, could not cover the compounds found with Tenax TA. In comparison to liquid extraction, thermal desorption prevents potential dilution of the compounds by the solvents and decreases sample preparation steps and the use for XAD-4 could have led to more recovered compounds. Moreover, now XAD-2 was also shown to be convenient for the adsorption of volatiles and thermal desorption [26]. Notably, in thermal desorbed TD tubes filled with two materials we found almost all of the compounds detected in the thermal desorbed tubes filled with only Tenax TA and additionally the compounds from XAD-4. Hence, the combination of two adsorbents is beneficial for capturing a broader spectrum of substances compared to the use of one material. Depending on the used materials and their affinity to specific substances, different substance classes will be captured in various amounts. For the combination of two adsorbent materials, temperature programs for cleaning and GC-MS analysis need to be adjusted to the specific temperature stability thresholds of the applied adsorbent materials to avoid degradation [26].

### Handling and feasibility

The decision on method selection and establishment for a lab is driven by various factors influencing the feasibility of a study. Not only the proper performance of the method analyzing the anticipated scope of compounds, but also soft criteria such as costs of purchase and consumables, expenditure of preparation and analysis time play an important role (see Table 4). From a cost perspective, the use of cotton swabs is the most affordable procedure with respect to acquisition as well as consumables compared to the other two methods. After precleaning, cotton swabs can be used in the field without further on-site preparation, the swabs are very easy to handle and sampling is quick. Even so, a direct contact with the animal body is needed for sampling, which will frequently be impossible for free-ranging animals without anesthesia, and samples require storage conditions of– 80°C, which is additionally challenging particular when transferring from the field to the lab is required.

**Table 4:** Comparison of key properties influencing the feasibility of the sampling methods.

Parameters	Cotton swabs	Mobile GC-MS	TD tubes
Purchase costs devices	Low	Intermediate	High (additional sampler required)
Acquisition costs: sampling material/consumables	Low/low	Intermediate/high	High/intermediate
Preparation time: prior in the lab/on-site in the field	Cleaning 1 h/none	None/cleaning 0.5 h	Cleaning 4 h/none
Field mobility	Light weight, no transport restriction	19 kg device, battery run time 2 h	Steel tubes recommended, relatively light weight, sampling pump weight 2.5 kg, battery run time 14 h
Sampling duration	20 sec per swab	Max. 1 min/100 mL	Max. 1 min/1000 mL
Maximum sample frequency	One swab per min	One in 15 min	One per min
Feasibility	Direct contact	Close distance	Variable distance
Sample storage	In glass vials at -80°C	No storage	At ambient temperature
Time until data acquisition	Transport duration and extraction (1 h)	Immediately	Transport duration and extraction (1 h)
Sample analysis	Headspace or liquid injection after solvent extraction	Thermal desorption immediately after sampling	Thermal desorption in GC-MS or liquid injection after solvent extraction
Target substances	semi-VOCs, nonVOCs	VOCs	VOCs, semi-VOCs, nonVOCs
Contaminations/sample background	high	low	Low

Certainly, the on-site analysis of body odor samples immediately after sampling with mobile GC-MS devices is an interesting option for field workers. Furthermore, no direct contact to the animals is needed because the surrounding air is sampled. However, the instrument needs preparation directly before sampling and prior to every new sample, hence, sampling can proceed, at the soonest, after the first sample’s analysis time plus a few min for instrument cleaning and equilibration. The mobility of the instrument (portable as backpack) might furthermore be restricted by the weight of 19 kg. Compared to the TD tubes which are connected to a small pump of 2.5 kg weight (BiVOC2), the sampling duration with the mobile GC-MS is much longer to get an appropriate same sample volume due to the lower maximum flow (see Table 4). Moreover, sequential sampling after interruption of a sampling run is not possible, which would be beneficial for free-ranging animals that are more likely to move away before the intended sample volume is reached unless they are trained to do so.

Independent from the adsorbent materials, sampling with TD tubes is a very convenient, versatile and at the same time efficient method for body odor collection. The tubes are precleaned in the laboratory and can be used without further on-site preparation. The sampling using a small pump is very flexible. Using the maximal flow (~ 1 L/min with BiVOC2) a sample of 500 mL can be taken within 30 s. Sampling with a high flow rate could cause a breakthrough of the compounds. Woolfenden [57] summarized that the sampling flow should not exceed 200 mL/min except for sampling durations below 10 min. Thus, we used the maximal flow to lower the handling duration during sampling for the animals. Otherwise, experiments could be carried out prior sampling to investigate the breakthrough threshold depending on sampling flow, volume and duration. If the target animals/species is tolerant for approaches (as the pump is not a matter of disturbance), sampling can be done with a lower flow to avoid a breakthrough and to increase the interaction time between the adsorbent and the volatiles, if required by the adsorbent. One body odor sample can also be divided into multiple sub-samples with shorter durations to get a more intense sample from moving animals (Weiß et al. under revision). Additionally, the tubes can be directly held in the hand or be fixed at a stick to extend the distance to the animal during sampling and the tubes can be stored at ambient temperature. Analyzing TD tube samples with a lab GC-MS is approximately similarly costly as direct sampling with the mobile GC-MS when considering a long-term use. The background noise in the chromatograms is as low as for the mobile GC-MS, but the identification of compounds is improved because of the better GC-MS resolution and the flexible options of data evaluation through interchangeable data formats.

### Conclusions

Considering all advantages and disadvantages of the compared methods, the most suitable sampling method for body odor of mammals appears to be the adsorption to TD tubes. This sampling method captures volatile compounds best, provides high-quality samples and is flexible because the most appropriate adsorbent or a combination can be selected. Additionally, the sampling procedure can be adjusted to the given study conditions very easily. Certainly, cotton swabs can be used for sampling chemicals from the skin or fur of a mammal, but collected compounds mostly feature higher boiling points, which may be precursors of breakdown products, for instance, due to the metabolism of skin bacteria and therefore contribute indirectly to body odor. Mobile GC-MS can be used to analyze the more abundant volatiles in the body odor of mammals within the given sensitivity limitations of the device. However, pilot studies are recommended beforehand. Finally, further studies on the body odor of mammals should take the variety of usable methods into account, choose the most appropriate one based on the study parameters as well as requirements and interpret results within the possibilities and limitations of the selected method.

## Supporting information

S1 Fig Chromatograms of all three body odor sampling methods. A: cotton swab, B: TD tube MIX, C: mobile GC-MS. Samples were taken from same female common marmoset consecutively at the same sampling day, dashed line marks the threshold between volatile (left part) and semi- or non-volatile (right part) compounds, whereas in part C all compounds are volatile.
(PDF)

S1 Table Sampling dates of samples taken with all five sampling materials from the six animals. (PDF)

S2 Table Overview of all confirmed or identified compounds. Confirmed (“conf”) compounds of TD tube Tenax, XAD and Mix, as well as the identified mobile GC-MS compounds, and additionally the biggest compounds of cotton swabs, TD tubes Tenax, XAD and Mix with CAS number (* NIST number, if no CAS number was available), boiling point (bp, in °C at 760 mmHg, www.chemspider.com), sample set (Set), retention time (RT), mean match of substance suggestions if not confirmed (Match), retention time of corresponding peaks in TD tubes Mix (TD), substance classification (Class), origin category (origin, exo = exogenous, pot endo = potentially endogenous, pot met = potentially metabolized) and references to animal odor studies (if not assign to other origin).
(PDF)
