# Supplementary material for: Different methods for volatile sampling in mammals
Source: PLoS One. 2017 Aug 25;12(8):e0183440. doi: 10.1371/journal.pone.0183440 (PMC5571906; doi:10.1371/journal.pone.0183440)

SUPPLEMENT

S1 Fig. Chromatograms of all three body odour sampling methods. A: cotton swab, B: TD tube MIX, C: mobile GC-MS. Samples were taken from same female common marmoset consecutively at the same sampling day, dashed line marks the threshold between volatile (left part) and semi- or non-volatile (right part) compounds, whereas in part C all compounds are volatile.

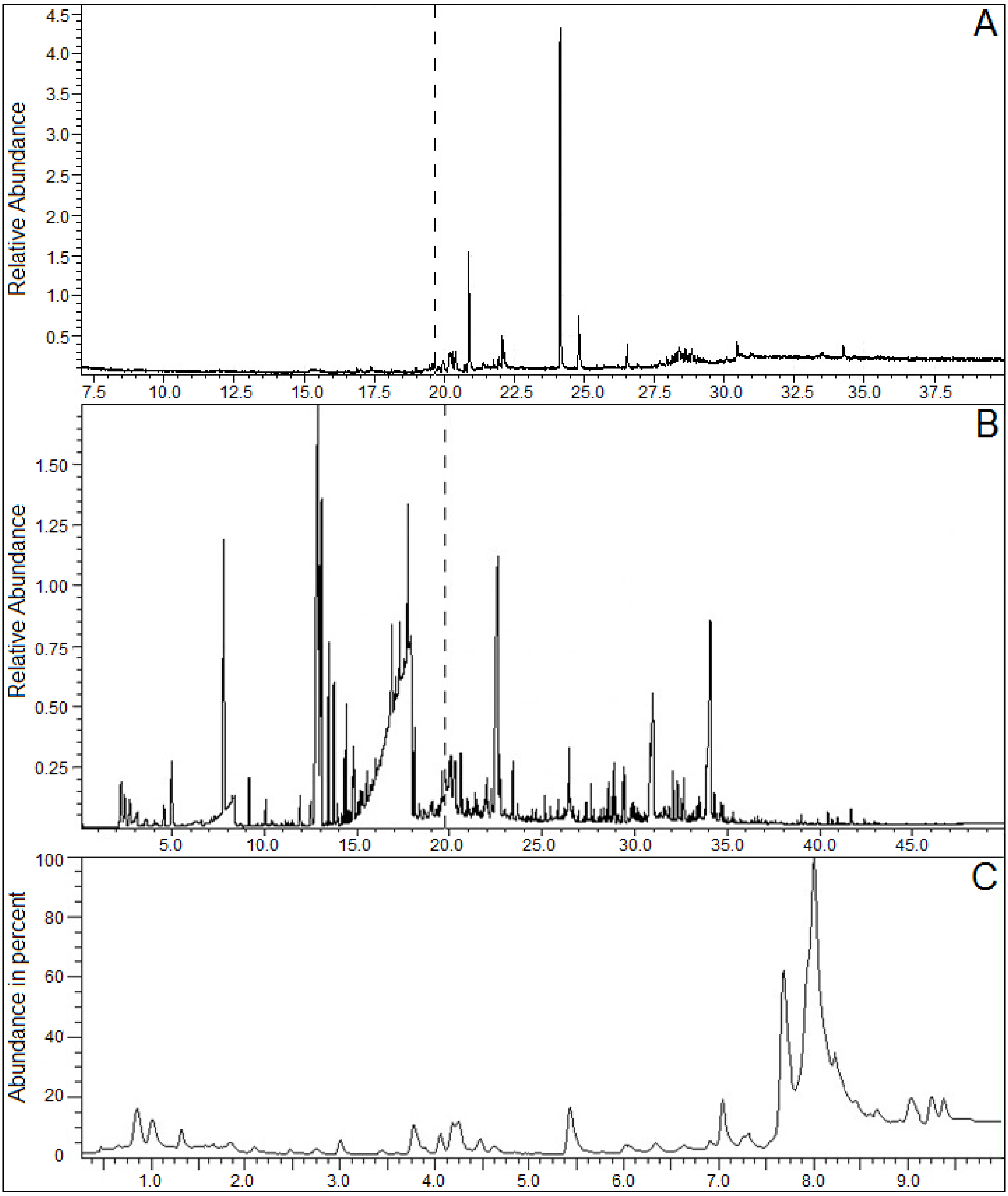

Supplement: S1 Fig — A: cotton swab, B: TD tube MIX, C: mobile GC-MS. Samples were taken from same female common marmoset consecutively at the same sampling day, dashed line marks the threshold between volatile (left part) and semi- or non-volatile (right part) compounds, whereas in part C all compounds are volatile. (PDF) [file pone.0183440.s001.pdf]
