# Supplementary material for: Different methods for volatile sampling in mammals
Source: PLoS One. 2017 Aug 25;12(8):e0183440. doi: 10.1371/journal.pone.0183440 (PMC5571906; doi:10.1371/journal.pone.0183440)
Supplement: S1 Table — (PDF) [file pone.0183440.s002.pdf]

SUPPLEMENT

S1 Table. Sampling dates of samples taken with all five sampling materials from the six animals.

	Cotton swabs	TD tubes Mix	Mobile GC-MS	TD tubes Tenax	TD tubes XAD
ID 1	25.06.2015	25.06.2015	25.06.2015	17.07.2015*	17.07.2015*
ID 1	22.07.2015	22.07.2015	22.07.2015	22.07.2015	---
ID 2	28.02.2015	28.02.2015	28.02.2015	17.07.2015*	17.07.2015*
ID 2	02.03.2015	02.03.2015	02.03.2015	21.07.2015*	21.07.2015*
ID 3	02.03.2015	02.03.2015	02.03.2015	17.07.2015*	17.07.2015*
ID 3	03.03.2015	03.03.2015	03.03.2015	21.07.2015*	21.07.2015*
ID 4	25.06.2015	25.06.2015	25.06.2015	22.07.2015*	---
ID 4	21.07.2015	21.07.2015	21.07.2015	21.07.2015	21.07.2015
ID 5	21.07.2015	21.07.2015	21.07.2015	21.07.2015	21.07.2015
ID 5	22.07.2015	22.07.2015	22.07.2015	22.07.2015	22.07.2015
ID 6	04.03.2015	04.03.2015	04.03.2015	17.07.2015*	17.07.2015*
ID 6	22.07.2015	22.07.2015	22.07.2015	22.07.2015	22.07.2015

* Samples taken from different days than other samples and thus are parallel only with regard to animal ID.
