# Supplementary material for: Different methods for volatile sampling in mammals
Source: PLoS One. 2017 Aug 25;12(8):e0183440. doi: 10.1371/journal.pone.0183440 (PMC5571906; doi:10.1371/journal.pone.0183440)
Supplement: S2 Table — Confirmed (“conf”) compounds of TD tube Tenax, XAD and Mix, as well as the identified mobile GC-MS compounds, and additionally the biggest compounds of cotton swabs, TD tubes Tenax, XAD and Mix with CAS number (* NIST number, if no CAS number was available), boiling point (bp, in °C at 760 mmHg, www.chemspider.com), sample set (Set), retention time (RT), mean match of substance suggestions if not confirmed (Match), retention time of corresponding peaks in TD tubes Mix (TD), substance classification (Class), origin category (origin, exo = exogenous, pot endo = potentially endogenous, pot met = potentially metabolized) and references to animal odor studies (if not assign to other origin). (PDF) [file pone.0183440.s003.pdf]

SUPPLEMENT

S2 Table. Overview of all confirmed or identified compounds. Confirmed ("conf") compounds of TD tube Tenax, XAD and Mix, as well as the identified mobile GC-MS compounds, and additionally the biggest compounds of cotton swabs, TD tubes Tenax, XAD and Mix with CAS number (* NIST number, if no CAS number was available), boiling point (bp, in °C at 760 mmHg, www.chemspider.com), sample set (Set), retention time (RT), mean match of substance suggestions if not confirmed (Match), retention time of corresponding peaks in TD tubes Mix (TD), substance classification (Class), origin category (origin, exo = exogenous, pot endo = potentially endogenous, pot met = potentially metabolized) and references to animal odour studies (if not assign to other origin). References are shown in a reference style with detailed author and year information for a better overview.

Substance	CAS-Nr.	B.p. (°C)	Set	RT	Match	TD	Class	Origin	Odour references
Cyclohexane, methyl-	108-87-2	101.1±3.0	Mobile	2.06	878	4.54	alkane	pot metab	De Preter et al. 2009; Costello et al. 2014; Marneweck et al. 2017
Toluene	108-88-3	110.6±3.0	Mobile	2.29	838	5.11	alkene	exo	Bernier et al. 2000
Heptane, 2-methyl-	592-27-8	117.8±3.0	Mobile	2.35	940	4.97	alkane	pot metab	Costello et al. 2014
Hexanal	66-25-1	127.9±3.0	Mobile	2.45	946	5.38	alkanal	pot endo	Burger et al. 1999; Curran et al. 2005; Burger 2005; Garner et al. 2007; Costello et al. 2014
Propanoic acid, propyl ester	106-36-5	121.9±3.0	Mobile	2.57	836	5.91	ester	pot endo	Garner et al. 2007
Ethylbenzene	100-41-4	136.2±3.0	Mobile	3.54	738	7.13	aromatic hydrocarbon	exo	Bernier et al. 2000; Costello et al. 2014; solvent (Marcillo et al. in press)
Nonane	111-84-2	151.7±3.0	Mobile	4.49	956	8.40	alkane	pot metab	Burger et al. 1999; Bernier et al. 2000; Curran et al. 2005; Curran et al. 2010; Burger 2005; Costello et al. 2014
Benzene, (1-methylethyl)-	98-82-8	152.4	Mobile	5.02	971	8.69	aromatic hydrocarbon	exo	Adsorbent artifact (Marcillo et al. in press)
5-Hepten-2-one, 6-	110-93-0	173.3±9.0	Mobile	6.03	941	10.03	ketone	pot metab	Bernier et al. 2000;

methyl-									Curran et al. 2005; Garner et al. 2007; Gallagher et al. 2008; Charpentier et al. 2012; Costello et al. 2014
D-Limonene	5989-27-5	175.4±20.0	Mobile	7.04	800	11.50	terpene	pot metab	Costello et al. 2014
Acetophenone	98-86-2	202	Mobile	7.19	927	12.18	ketone, aromatic	exo	Burger 2005; Garner et al. 2007; Costello et al. 2014
Undecane	1120-21-4	196.3±3.0	Mobile	8.15	920	14.49	alkane	exo	Curran et al. 2005; Garner et al. 2007; Curran et al. 2010; Setchell et al. 2010; Costello et al. 2014
Dodecane	112-40-3	216.1±3.0	Mobile	9.24	864	15.99	alkane	pot endo	Burger et al. 1999; Curran et al. 2005; Garner et al. 2007; Curran et al. 2010; Costello et al. 2014
Butanal	123-72-8	077.6±3.0	Tenax	6.35	conf	---	alkanal	pot endo	Curran et al. 2005; Garner et al. 2007; Curran et al. 2010
n-Propyl acetate	109-60-4	101.4±3.0	Tenax	7.21	conf	---	ester	pot metab	Flavour agent (Sabatini and Marsilio 2008)
Isobutanol	78-83-1	105±8.0	Tenax	7.26	conf	3.39	alcohol	pot endo	Solvent
1-Butanol	71-36-3	117.7±3.0	Tenax	8.18	conf	5.09	alcohol	pot endo	Garner et al. 2007; Gallagher et al. 2008; Costello et al. 2014
2,3-Pentanedione	600-14-6	108	Tenax	9.45	conf	4.57	ketone	pot metab	Garner et al. 2007; Costello et al. 2014
1-Pentanol	71-41-0	138.5±3.0	Tenax	12.62	conf	7.49	alcohol	pot metab	Garner et al. 2007
2,3-Butanediol	513-85-9	180.7	Tenax	14.20	conf	11.44	alcohol	pot endo	Costello et al. 2014
Butanoic acid	107-92-6	164.3±3.0	Tenax	14.30	conf	10.25	carboxylic acid	pot endo	Burger et al. 1999; Smith et al. 2001; Burger 2005;

									Garner et al. 2007; Gallagher et al. 2008; Costello et al. 2014
2-Hexanone, 5-methyl-	110-12-3	144	Tenax	17.09	conf	7.66	ketone	pot metab	Garner et al. 2007; Costello et al. 2014
2-Heptanone	110-43-0	151.2±3.0	Tenax	18.62	conf	7.66	ketone	pot metab	Garner et al. 2007; Costello et al. 2014
1-Heptanol	111-70-6	176.9±3.0	Tenax	22.22	conf	11.32	alcohol	pot metab	Garner et al. 2007; Costello et al. 2014
1-Octen-3-one	4312-99-6	177±9.0	Tenax	22.53	conf	11.33	ketone	pot endo	Costello et al. 2014
2-Heptanone, 6-methyl-	928-68-7	167	Tenax	23.07	conf	10.18	ketone	pot metab	Charpentier et al 2012; Costello et al. 2014
Eucalyptol	470-82-6	174±8.0	Tenax	24.88	conf	10.03	alcohol, terpene	pot metab	Costello et al. 2014
1-Nonanol	143-08-8	211.6±3.0	Tenax	29.83	conf	14.89	alcohol	pot metab	Garner et al. 2007; Charpentier et al. 2012; Costello et al. 2014
Nonanoic acid	112-05-0	254.9±3.0	Tenax	32.99	conf	17.63	carboxylic acid	pot metab	Bernier et al. 2000; Curran et al. 2005; Charpentier et al. 2012; Costello et al. 2014; Marneweck et al. 2017
2,3-Hexanedione	3848-24-6	125±9.0	XAD	6.35	conf	5.30	ketone	pot endo	Garner et al. 2007; Costello et al. 2014
Heptanoic acid, ethyl ester	106-30-9	188.3±3.0	XAD	15.77	conf	11.59	ester	pot metab	Garner et al. 2007; Costello et al. 2014
Decanoic acid, ethyl ester	110-38-3	242.5±3.0	XAD	20.50	conf	16.36	ester	pot endo	Charpentier et al. 2012; Costello et al. 2014
Dodecanal	112-54-9	242.2±3.0	XAD	20.77	conf	16.32	alkanal	pot endo	Bernier et al. 2000; Garner et al. 2007; Gallagher et al. 2008; Costello et al. 2014;
2,3-Butanedione	431-03-8	088	Mix	2.55	conf	---	ketone	pot endo	Garner et al. 2007; Costello et al. 2014
Triethylamine	121-44-8	090.5±8.0	Mix	3.12	conf	---	alkylamine	exo	Food compound (Yannai 2003)

Furan, 2-ethyl-	3208-16-0	094.5±9.0	Mix	3.19	conf	---	alkane	pot metab	Food compound (Yannai 2003)
Pyrimidine	289-95-2	122.4±9.0	Mix	3.49	conf	---	Pyrimidine	pot endo	Costello et al. 2014
Butanoic acid, ethyl ester	105-54-4	122.4±3.0	Mix	4.05	conf	---	ester	pot endo	Costello et al. 2014
Acetic acid, butyl ester	123-86-4	126.6±3.0	Mix	4.19	conf	---	ester	pot endo	Costello et al. 2014
3-Heptanone	106-35-4	148.5±3.0	Mix	5.05	conf	---	ketone	pot metab	Garner et al. 2007; Costello et al. 2014
2-Heptanol	543-49-7	159.4±3.0	Mix	5.30	conf	---	alcohol	pot metab	Food compound (Yannai 2003)
Acetic acid, pentyl ester	628-63-7	149.9±3.0	Mix	5.45	conf	---	ester	pot endo	Costello et al. 2014
n-Nonane	111-84-2	151.7±3.0	Mix	5.51	conf	---	alkane	pot metab	Costello et al. 2014
Hexanoic acid, methyl ester	106-70-7	149.8±3.0	Mix	5.61	conf	---	ester	pot endo	Curran et al. 2005; Charpentier et al. 2012; Costello et al. 2014
Phenol	108-95-2	181.8	Mix	6.37	conf	---	alcohol, aromatic	exo	Bernier et al. 2000; Smith et al 2001; Burger 2005; Curran et al. 2005; Garner et al. 2007; Gallagher et al. 2008; Curran et al. 2010; Costello et al. 2014;
Acetic acid, hexyl ester	142-92-7	171.5±3.0	Mix	6.90	conf	---	ester	pot endo	Costello et al. 2014
7-Octen-2-ol, 2,6-dimethyl-	18479-58-8	188.4	Mix	7.85	conf	---	alcohol	pot endo	Garner et al. 2007; Gallagher et al. 2008; Costello et al. 2014
Heptanoic acid	111-14-8	222.6±3.0	Mix	7.85	conf	---	carboxylic acid	pot metab	Bernier et al. 2000; Burger 2005; Garner et al. 2007; Costello et al. 2014
2-Nonanone	821-55-6	193.5±3.0	Mix	8.07	conf	---	ketone	pot metab	Garner et al. 2007; Charpentier et al 2012; Costello et al. 2014
1,6-Octadien-3-ol, 3,7-dimethyl-	78-70-6	198.5	Mix	8.28	conf	---	alcohol	pot metab	Burger 2005; Garner et al. 2007;

									Gallagher et al. 2008; Costello et al. 2014
2-Butanedioic acid (*Z*)-, diethyl ester	141-05-9	214	Mix	9.02	conf	---	ester	exo	Additive and intermediate for plastics
Benzaldehyde, 4-methoxy-	123-11-5	248	Mix	10.31	conf	---	aldehyde, aromatic	pot metab	Plant compound (Hirvi et al 1981); XAD contaminant (Marcillo et al. in press)
Alpha-Ionone	127-41-3	257.6	Mix	12.90	conf	---	ketone, terpene	pot metab	Penn et al. 2007; Costello et al. 2014
Beta-Ionone	14901-07-6	254.8	Mix	13.64	conf	---	ketone, terpene	pot metab	Costello et al. 2014
Hexane, 3,3-dimethyl-	563-16-6	111.1±7.0	Cotton	18.15	797	---	alkane	pot metab	Food compound (Yannai 2003)
Octane, 2,7-dimethyl-	1072-16-8	158.8±7.0	Cotton	19.58	815	---	alkane	pot metab	Charpentier et al. 2012
Pentadecanoic acid, 14-methyl-, methyl ester	5129-60-2	311.8±10.0	Cotton	20.30	778	---	ester	pot metab	Setchell et al. 2010; Charpentier et al. 2012
Oxalic acid, allyl nonyl ester	* 309237	323.3±11.0	Cotton	21.26	776	---	ester	pot metab	Plant compound (Zeidan et al. 2015)
Nonane, 4-methyl-5-propyl-	62185-55-1	216±7.0	Cotton	21.77	799	---	alkane	pot metab	Charpentier et al. 2012
Hexadecane	544-76-3	286.6±3.0	Cotton	22.95	793	---	alkane	pot metab	Bernier et al. 2000; Burger 2005; Curran et al. 2005; Garner et al. 2007; Costello et al. 2014
Sulfurous acid, butyl decyl ester	* 309177	344.9±11.0	Cotton	24.00	793	---	ester	NA	Instable (McCormack and Lawes 2000)
Sulfurous acid, 2-ethylhexyl isohexyl ester	* 309190	335.9±11.0	Cotton	24.34	793	---	ester	NA	Instable (McCormack and Lawes 2000)
Di(2-ethylhexyl) adipate	103-23-1	374.4	Cotton	24.80	888	---	ester	pot metab	Plasticiser (Gooch 2011)
Sulfurous acid, hexyl pentadecyl ester	* 309137	446.8±14.0	Cotton	24.90	770	---	ester	NA	Instable (McCormack and Lawes 2000)
Sulfurous acid, 2-propyl undecyl ester	* 309122	340.3±11.0	Cotton	25.56	726	---	ester	NA	Instable (McCormack and Lawes 2000)
Nonadecane	629-92-5	330.1±5.0	Cotton	25.87	810	---	alkane	pot metab	Garner et al. 2007;

									Costello et al. 2014
Cholest-5-en-3-ol (3β)-, 4-methylbenzenesulfonate	1182-65-6	617.5±34.0	Cotton	29.24	512	---	steroid ester	pot metab	Charpentier et al. 2012
Cholest-5-en-3-ol (3β)-, acetate	604-35-3	493.3±24.0	Cotton	29.24	532	---	steroid ester	pot endo	Charpentier et al. 2012
Cholesta-4,6-dien-3-ol, (3β)-	14214-69-8	492.9±14.0	Cotton	30.30	588	---	steroid	pot endo	Charpentier et al. 2012
Methane, chloro-	74-87-3	-24.5±3.0	Mix	2.22	964	---	alkane	exo	Costello et al. 2014
Acetaldehyde	75-07-0	018.6±3.0	Mix	2.26	903	---	aldehyde	pot endo	Costello et al. 2014
Isopropanol	67-63-0	073±3.0	Mix	2.51	903	---	alcohol	pot endo	Solvent and disinfectant
Acetic acid	64-19-7	117.1±3.0	Mix	3.15	930	---	carboxylic acid	pot endo	Bernier et al. 2000; Smith et al 2001; Burger 2005; Gallagher et al. 2008; Charpentier et al. 2012; Costello et al. 2014
2-Pentanol, 4-methyl-	108-11-2	133.5±8.0	Mix	5.03	913	---	alcohol	pot endo	Gingell et al. 2003
1,3,5,7-Cyclooctatetraene	629-20-9	140.5	Mix	7.81	820	---	alkene, aromatic	pot metab	Costello et al. 2014
4-Ethylbenzoic acid	619-64-7	270.3±19.0	Mix	19.99	880	---	carboxylic acid, aromatic	pot metab	Adsorbent artifact (Marcillo et al. in press)
Tetradecane	629-59-4	253.9±3.0	Mix	20.76	901	---	alkane	pot metab	Bernier et al. 2000; Burger 2005; Curran et al. 2005; Garner et al. 2007; Curran et al. 2010; Costello et al. 2014
Benzoic acid, 4-acetyl-, methyl ester	3609-53-8	295.6±23.0	Mix	21.26	822	---	ester, aromatic	pot metab	Charpentier et al. 2012
Pentadecane	629-62-9	270.6±3.0	Mix	22.50	913	---	alkane	pot metab	Bernier et al. 2000; Setchell et al. 2010; Costello et al. 2014
1(2H)-Naphthalenone, 3,4-dihydro-2-(phenylmethyl)-	27019-08-5	381.4±12.0	Mix	29.63	679	---	ketone, aromatic	pot metab	Sorbent degradation (Marcillo et al. in press)
n-Hexadecanoic acid	57-10-3	340.6±5.0	Mix	31.12	918	---	carboxylic acid	pot endo	Scordato et al. 2007;

									Charpentier et al 2012
Octadecanoic acid	57-11-4	359.4±5.0	Mix	34.12	893	---	carboxylic acid	pot endo	Burger et al. 1999; Bernier et al. 2000; Burger 2005; Scordato et al. 2007; Costello et al. 2014
1,3-Propanedione,2,2-dimethyl-1,3-diphenyl-	41169-42-0	402.7±28.0	Mix	35.31	826	---	ketone, aromatic	pot metab	Synthetic chemical (Bhowmick et al. 2002)
Hexacosane	630-01-3	412.2±8.0	Mix	41.43	875	---	alkane	pot metab	Costello et al. 2014
Acetone	67-64-1	046.5±3.0	Tenax	5.16	932	---	ketone	pot endo	Garner et al. 2007; Gallagher et al. 2008; Costello et al. 2014
n-Hexane	110-54-3	068.5±3.0	Tenax	6.24	936	---	alkane	pot metab	Gassett et al. 1996
2-Butanone	78-93-3	075.6±3.0	Tenax	6.62	922	---	ketone	pot metab	Garner et al. 2007; Costello et al. 2014
Acetic acid, anhydride with formic acid	2258-42-6	102.6±23.0	Tenax	7.47	839	---	ester	pot endo	Synthetic chemical (Pivovarenko and Khilya 1992)
1,3,5-Cycloheptatriene	544-25-2	116.5	Tenax	12.24	930	---	alkene	exo	Costello et al. 2014
Octane	111-65-9	126.4±3.0	Tenax	13.61	941	---	alkane	pot metab	Burger et al. 1999; Bernier et al. 2000; Burger 2005; Garner et al. 2007; Costello et al. 2014
Hydrazine, methyl-	60-34-4	087.5±9.0	XAD	5.60	771	---	hydrazine	exo	Natural carcinogen (Havender and Coulombe 1996)
Methyl methacrylate	80-62-6	100.3	XAD	6.50	803	---	ester	exo	Costello et al. 2014
Hydroxylamine	7803-49-8	056.5±9.0	XAD	6.76	710	---	hydroxylamine	pot metab	Food compound (Yannai 2003)
2,2-Dimethoxybutane	3453-99-4	099.8±8.0	XAD	7.05	859	---	ether	pot metab	Plant compound (Cock and Kalt 2012)
2-Propenoic acid, ethyl ester	140-88-5	099.5	XAD	7.22	875	---	ester	pot endo	Costello et al. 2014
Hydrazine	302-01-2	113.5±9.0	XAD	7.40	882	---	hydrazine	exo	Costello et al. 2014

Benzoic acid, methyl ester	93-58-3	199.5	XAD	18.20	869	---	ester, aromatic	pot metab	Gassett et al. 1996
3-Methylglutaconic acid, diethyl ester	55887-63-3	251.4±15.0	XAD	19.77	809	---	ester	pot metab	Bouatra et al. 2013
Ethanol	64-17-5	072.6±3.0	Tenax/ XAD	5.27/ 5.9	914/ 831	---	alcohol	pot endo	Garner et al. 2007; Costello et al. 2014
1-Propanol	71-23-8	095.8±3.0	Mobile/ Tenax	1.00/ 6.48	935/ 921	2.78	alcohol	pot endo	Garner et al. 2007; Costello et al. 2014
Pyrazine, 2,5-dimethyl-	123-32-0	155	Mobile/ Tenax	4.38/ 19.54	965/ 915	8.05	pyrazine	pot metab	Smith et al. 2001; Costello et al. 2014
Octanal	124-13-0	163.4	Tenax/ Mix	23.60/ 6.72	conf	10.47	alkanal	pot endo	Bernier et al. 2000; Burger 2005; Curran et al. 2005; Garner et al. 2007; Gallagher et al. 2008; Costello et al. 2014
Benzaldehyde	100-52-7	178.7	Mobile/ Mix/ Tenax	5.27/ 6.03/ 23.74	808/ conf/ 891	9.16	aldehyde, aromatic	pot metab	Bernier et al. 2000; Smith et al 2001; Burger 2005; Curran et al. 2005; Curran et al. 2010; Costello et al. 2014
Nonanal	124-19-6	190.8±3.0	Mobile/ Tenax/ XAD	8.00/ 27.53/ 13.92	908/ conf/ 896	13.92	alkanal	pot metab	Bernier et al. 2000; Burger et al. 1999; Costello et al. 2014; Curran et al. 2005; Curran et al. 2010; Gallagher et al. 2008; Garner et al. 2007
Hexanoic acid	142-62-1	204.6±3.0	Tenax/ Mix	23.04/ 6.39	conf	13.42	carboxylic acid	pot endo	Bernier et al. 2000; Burger 2005; Garner et al. 2007; Gallagher et al. 2008; Setchell et al. 2010; Costello et al. 2014

Decanal	112-31-2	209.0±3.0	Mobile/ Tenax/ XAD	9.17/ 31.00/ 18.13	774/ conf/ conf	15.63	alkanal	pot endo	Bernier et al. 2000; Curran et al. 2005; Garner et al. 2007; Gallagher et al. 2008; Curran et al. 2010; Costello et al. 2014; Marneweck et al. 2017
Benzoic acid, ethyl ester	93-89-0	211.7±8.0	XAD/ Mix	17.80/ 9.23	conf	14.66	ester, aromatic	pot metab	Setchell et al. 2010; XAD contaminant
Undecanal	112-44-7	226.1±3.0	Tenax/ Mix	34.11/ 11.24	conf	15.99	alkanal	pot metab	Curran et al. 2005; Penn et al. 2007; Costello et al. 2014
Octanoic acid	124-07-2	239.3±3.0	Tenax/ Mix	29.85/ 9.28	conf	15.97	carboxylic acid	pot endo	Burger et al. 1999; Bernier et al. 2000; Burger 2005; Garner et al. 2007; Curran et al. 2010; Charpentier et al. 2012; Costello et al. 2014

References

Bernier, U.R., Kline, D.L., Barnard, D.R., Schreck, C.E., and Yost, R.A. 2000. Analysis of Human Skin Emanations by Gas Chromatography/Mass Spectrometry. 2. Identification of Volatile Compounds That Are Candidate Attractants for the Yellow Fever Mosquito (*Aedes aegypti*). Anal Chem. 72:747–756.

Bhowmick, K.C., Prasad, K.R.K., and Joshi, N.N. 2002. Synthesis and resolution of 2,2-dimethyl-1,3-diphenyl-1,3-propanediol, a new C2-symmetric and conformationally rigid acyclic diol. Tetrahedron Asymmetry. 13:851–855.

Bouatra, S., Aziat, F., Mandal, R., Guo, A.C., Wilson, M.R., Knox, C., Bjorndahl, T.C., Krishnamurthy, R., Saleem, F., Liu, P., et al. 2013. The Human Urine Metabolome. PLoS ONE. 8:1–28.

Burger, B.V. 2005. Mammalian Semiochemicals. Top Curr Chem. 240:231–278.

Burger, B.V., Greyling, J., and Spies, H.S.C. 1999. Mammalian Exocrine Secretions. XIV: Constituents of Preorbital Secretion of Steenbok, Raphicerus campestris. J Chem Ecol. 25:2099–2108.

Charpentier, M.J.E., Barthes, N., Proffit, M., Bessière, J.-M., and Grison, C. 2012. Critical thinking in the chemical ecology of mammalian communication: roadmap for future studies. Funct Ecol. 26:769–774.

Cock, I.E., and Kalt, F.R. 2012. Gas chromatography-mass spectroscopy analysis of a Xanthorrhoea johnsonii leaf extract displaying apparent anaesthetic effects. J Nat Pharm. 3:78–88.

Costello, B. de L., Amann, A., Al-Kateb, H., Flynn, C., Filipiak, W., Khalid, T., Osborne, D., and Ratcliffe, N.M. 2014. A review of the volatiles from the healthy human body. J Breath Res. 8:014001.

Curran, A.M., Prada, P.A., and Furton, K.G. 2010. The Differentiation of the Volatile Organic Signatures of Individuals Through SPME-GC/MS of Characteristic Human Scent Compounds. J Forensic Sci. 55:50–57.

Curran, A.M., Rabin, S.I., Prada, P.A., and Furton, K.G. 2005. Comparison of the volatile organic compounds present in human odor using spme-GC/MS. J Chem Ecol. 31:1607–1619.

De Preter V, Van Staeyen G, Esser D, Rutgeerts P, Verbeke K. Development of a screening method to determine the pattern of fermentation metabolites in faecal samples using on-line purge-and-trap gas chromatographic–mass spectrometric analysis. J Chromatogr A. 2009 Feb 27;1216(9):1476–83.

Gallagher, M., Wysocki, C.J., Leyden, J.J., Spielman, A.I., Sun, X., and Preti, G. 2008. Analyses of volatile organic compounds from human skin. Br J Dermatol. 159:780–791.

Garner, C.E., Smith, S., Lacy Costello, B. de, White, P., Spencer, R., Probert, C.S.J., and Ratcliffe, N.M. 2007. Volatile organic compounds from feces and their potential for diagnosis of gastrointestinal disease. FASEB J. 21:1675–1688.

Gassett, J.W., Wiesler, D.P., Baker, A.G., Osborn, D.A., Miller, K.V., Marchinton, R.L., and Novotny, M. 1996. Volatile compounds from interdigital gland of male white-tailed deer (Odocoileus virginianus). J Chem Ecol. 22:1689–1696.

Gingell, R., Régnier, J.-F., Wilson, D.M., Guillaumat, P.-O., and Appelqvist, T. 2003. Comparative metabolism of methyl isobutyl carbinol and methyl isobutyl ketone in male rats. Toxicol Lett. 136:199–204.

Gooch, J.W. 2011. Encyclopedic dictionary of polymers. New York: Springer Science+Business Media.

Havender, W.R., and Coulombe, R. 1996. Does Nature Know Best?: Natural Carcinogens and Anticarcinogens in America's Food. Am Cncl on Science, Health.

Hirvi, T., Honkanen, E., and Pyysalo, T. 1981. The aroma of cranberries. Z Für Lebensm-Unters Forsch. 172:365–367.

Marcillo A, Jakimovska V, Widdig A, Birkemeyer C. XAD-2–a useful alternative to Tenax TA for analysis of biogenic volatiles employing thermal desorption coupled with gas chromatography − mass spectrometry. J Chromatogr A [Internet]. in press; Available from: http://www.sciencedirect.com/science/article/pii/S002196731730972X

Marneweck, C., Jürgens, A., and Shrader, A.M. 2017. Dung odours signal sex, age, territorial and oestrous state in white rhinos. Proc R Soc B. 284:20162376.

McCormack, W.B., and Lawes, B.C. 2000. Sulfuric and Sulfurous Esters. In: Kirk-Othmer Encyclopedia of Chemical Technology. John Wiley & Sons, Inc. p.

Penn, D.J., Oberzaucher, E., Grammer, K., Fischer, G., Soini, H.A., Wiesler, D., Novotny, M.V., Dixon, S.J., Xu, Y., and Brereton, R.G. 2007. Individual and gender fingerprints in human body odour. J R Soc Interface. 4:331–340.

Pivovarenko, V.G., and Khilya, V.P. 1992. Mixed anhydride of acetic and formic acids in the synthesis of chromones. Chem Heterocycl Compd. 28:497–502.

Sabatini, N., and Marsilio, V. 2008. Volatile compounds in table olives (Olea Europaea L., Nocellara del Belice cultivar). Food Chem. 107:1522–1528.

Scordato, E.S., Dubay, G., and Drea, C.M. 2007. Chemical composition of scent marks in the ringtailed lemur (*Lemur catta*): glandular differences, seasonal variation, and individual signatures. Chem Senses. 32:493–504.

Setchell, J.M., Vaglio, S., Moggi-Cecchi, J., Boscaro, F., Calamai, L., and Knapp, L.A. 2010. Chemical composition of scent-gland secretions in an Old World monkey (*Mandrillus sphinx*): Influence of sex, male status, and individual identity. Chem Senses. 35:205–220.

Smith, T.E., Tomlinson, A.J., Mlotkiewicz, J.A., and Abbott, D.H. 2001. Female marmoset monkeys (*Callithrix jacchus*) can be identified from the chemical composition of their scent marks. Chem Senses. 26:449–458.

Yannai, S. 2003. Dictionary of Food Compounds with CD-ROM: Additives, Flavors, and Ingredients. Taylor & Francis.

Zeidan, S., Hijazi, A., Rammal, H., Bazzal, A.A., Annan, H., and Al-Rekaby, A.A.-A.N. 2015. Determination of the Total Phenolic content, Total Flavonoid Content, Antioxidant Activity and Some Bioactive compounds of the Lebanese Eryngium creticum L. Eur Chem Bull. 4:498–502.
